# The Role of *Foxp3*-Expressing Regulatory T Cells and T Helpers in Immunopathogenesis of Multidrug Resistant Pulmonary Tuberculosis

**DOI:** 10.1155/2012/931291

**Published:** 2012-05-10

**Authors:** E. G. Churina, O. I. Urazova, V. V. Novitskiy

**Affiliations:** SBEI-HPE “Siberian State Medical University of the Ministry of Health Care and Social Development of the Russian Federation”, Tomsk, Russia

## Abstract

Subpopulation structure of regulatory T cells and T helpers of peripheral blood in patients with newly diagnosed pulmonary tuberculosis depending on the clinical form of disease and sensitivity of *Mycobacterium tuberculosis* to antituberculosis drugs has been analyzed in this work. It has been shown that the leading part in immune suppression at infiltrative, dissemination, and fibrosis-cavity pulmonary tuberculosis is played by natural regulatory CD4^+^CD25^+^Foxp3^+^-T lymphocytes. Thus we estimate increase of their number in blood by drug-resistance and drug-susceptible patients. It has been demonstrated that in patients with fibrocavernous and infiltrative form of the disease and drug-resistant pulmonary tuberculosis the number of CD4^+^CD25^−^Foxp3^+^-regulatory T cells was increasing. In patients with infiltrative pulmonary tuberculosis, including multidrug-resistant *M. tuberculosis*, an increased number of CD3^+^CD4^+^CD25^−^ T helpers is determined by the pathogenic features of the development of the tuberculosis infection and is connected with the activation of Th1-dependent immune response. Reduction in the number of T-helpers in the blood of patients with dissemination and fibrosis-cavity pulmonary tuberculosis mediates inefficient implementation of cell-mediated protective immunity.

## 1. Introduction

Drug-resistant pulmonary tuberculosis (DR-TB) is a case of tuberculosis caused by *M. tuberculosis *strains (MBTs) which are drug resistant to the effect of antituberculosis drugs (ATDs). It is supposed that drug-resistance (DR) is, above all, connected with accumulation of mutations in *M. tuberculosis *genes [1]. Multidrug-resistant tuberculosis (MDR-TB) is a special form of drug-resistant TB. It develops in case of resistance of *M. tuberculosis* at least to isoniazid and rifampicin—the two most powerful ATDs [2]. The problem of multidrug resistance of a tuberculosis causative agent to ATD in newly detected patients has lately gained global importance [3, 4]. According to the data of World Health Organization (2010), based on the information received from 114 countries around the world, primary MDR of MBT comprises about 4% from all newly detected TB cases, whereas on the territory of the CIS countries (Russia, Belarus, Ukraine, Kazakhstan, Armenia, and Azerbaijan) this indicator is 3–6 times higher [2].

Increase in morbidity of cases with primary DR-TB in patients who earlier did not receive ATD is especially alarming [5, 6]. Primary DR-TB develops as a result of primary infection by drug-resistant *M. tuberculosis* strains. In some regions of Russia secondary (acquired) DR-TB to ATD among earlier treated patients reaches 88% [5, 7]. Besides, an unfavorable tendency towards the increase in specific gravity of polyresistance and the decrease in specific gravity of monoresistance to ATD is marked; that is, at present MDR-TB is encountered more frequently among TB patients than DR-TB [5, 6]. A serious problem is a rise in the number of cases of primary DR to the most active chemical drugs—isoniazid and rifampicin—which, in combination with resistance to other first-line ATD or without it, is classified as MDR-TB, whereas in combination with resistance to second-line drugs, including fluoroquinolones and one of the injectable drugs (such as kanamycin or capreomycin), it is classified as XDR-TB [3, 5].

Clinical treatment of patients with MDR-TB is 3 times less than of those with TB whose causative agent is sensitive to ATD, in other words, effectiveness of treatment of such patients, which is determined by the cease in bacterioexcretion, is 3 times lower in MDR-TB than in drug-resistant variants of the disease. Besides, the frequency of termination of selection of ATD-sensitive MBT in patients reaches 92,5%, whereas in cases of TB caused by resistant strains of the causative agent only 58,1% [5, 8]. Therefore, ATD-multiresistant MBT becomes the major component of TB morbidity and mortality, which poses a serious threat to the whole mankind [2–5].

It is obvious that the above-stated problem requires many-sided and integrated approaches to its solution, the main of which is studying of immunopathogenic processes accompanying the course of pulmonary tuberculosis. Nowadays it is commonly believed that the development and the progressive course of the tubercular infection are impossible without functional defects in the protective cell immunity system [9]. Enhanced proliferation and excessive activity of regulatory T cells, which tend to weaken the anti-infectious organism immunity, are at present viewed as one of the mechanisms of Th-1-dependent immune response suppression, aimed at elimination of pathogens of various nature [10–12]. From all the identified regulatory T cells (Treg) subpopulations, Treg, expressing intracellular transcription factor *Foxp3, *which is the most precise marker of regulatory T-lymphocyte identification, has today the most functional activity in the aspect of implementation of immunosuppressive mechanisms. It is worth mentioning that *Foxp3*-positive regulatory T cells can be both natural (formed in the course of antigen-dependent differentiation in the thymus) and induced in the periphery in the process of adaptive immune system [13, 14]. One of the major indicators of Treg functional activity is their suppression of proliferation of T-helper effector clones, which mediates T-cell anergy formation.

**The Objective of the Work**

To define the role of *Foxp3*-expressing regulatory T cells and T helpers in immunopathogenesis of multidrug-resistant pulmonary tuberculosis.

## 2. Materials and Methods

The diagnosis of pulmonary tuberculosis was put on the basis of the clinical picture of the disease as well as X-ray lungs examination and data of microscopic and bacteriological sputum tests. The causative agent of tuberculosis was detected by means of direct light microscopy of the sputum smear, with the use of Ziehl-Neelsen stain, by the method of fluorescence microscopy with the use of fluorochromes (auramine). For species identification of *M. tuberculosis *and definition of sensitivity to anti-TB chemodrugs (a method of absolute concentration), we conducted sputum culture on dense Lowenstein-Jensen and Finn-2 media.

### 2.1. Microbiological Research Methods

To determine drug resistance of *M. tuberculosis* to basic ATD (rifampicin (RIF), isoniazid (INH), streptomycin, and ethambutol), we used the traditional bacteriological method of absolute concentrations. To carry out microbiological tests, we collected sputum in sterile 50 mL plastic test tubes with hermetically sealed screw caps. After sputum decontamination and MTB concentrating, the washed MTB sediment was used for culture on dense Lowenstein-Jensen medium with further detection of MTB sensitivity to RIF, INH, streptomycin, and ethambutol using the bacteriological absolute concentration method.

Mononuclear cells of peripheral blood, which was taken in the quantity of 10 mL from the cubital vein on an empty stomach in the morning, before the course of specific anti-tubercular therapy, served as the material of the research. Mononuclear cells of peripheral blood were isolated by gradient centrifugation [15].

### 2.2. Isolation of Mononuclear Leucocytes from Whole Blood

Heparinized venous blood (25 units/mL) was kept at the temperature of 37°C for 30 min to separate plasma from erythrocytes. The obtained plasma was layered on Ficoll-urografin (*ρ* = 1077 g/cm^3^) density gradient (“MP Biomedical, LLC”, USA) in a 1 : 2 ratio and centrifuged at 1500 rev/min for 20 min. The resulting interphase ring consisting of a mixture of mononuclear cells was collected with a pipette and then thrice washed with RPMI-1640 medium (“Vektor,” Russia), which was supplemented with 100 mkg/mL of gentamicin and 5% inactivated fetal calf serum (“BioloT LLC”, Russia), being consistently resuspended and centrifuged once every 10 min at 1500 rev/min. When using the gradient with the above-stated density, 90–95% of all the isolated mononuclear cells were lymphocytes.

### 2.3. Determination of the Quantity of CD4^+^CD25^+^Foxp3^+^, CD4^+^CD25^+^FoxP3^−^, and CD4^+^CD25^−^FoxP3 Regulatory T Cells in Peripheral Blood

To define CD4, CD25 superficial receptors and *Foxp3 *intracellular marker of immunosuppression activity in peripheral blood lymphocytes, we used the method of laser three-color cytometry with the use of fluorescently labeled multichannel antibodies (MCAB). Staining of superficial (CD4, CD25) and intracellular (*Foxp3*) markers was conducted according to the protocol of the manufacturing company (“Becton Dickinson (BD),” USA).

**Course of Work**

After isolation, the mononuclear leucocytes were twice washed with phosphate-saline buffer (pH = 7,4) the amount of cells in the suspension was standardized to 10 × 10^6^ cells/mL. To stain superficial markers (CD4, CD25) of the peripheral blood leucocytes, 20 mcl of the corresponding fluorescently labeled MCAB was added to the suspension of mononuclear leucocytes: to CD4—FITC-labeled, to CD25—PE-Cy5-labeled (“Becton Dickinson (BD)”, USA) and then incubated at room temperature for 20 min, while providing protection from light. To stain the intracellular marker *Foxp3 *the process of cell permeabilization was conducted. For this we alternately added working solutions of standard buffers to every test tube: Human *FoxP3* Buffer A and Human *FoxP3* Buffer C from the set BD Pharmingen Human *FoxP3* Buffer Set Cat. No. 560098. The buffers were diluted according to the instruction (cat. No. 560098). Leucocytes have been incubated for 30 min in a dark place at room temperature. Then the cells were twice washed with 2 mL of phosphate-saline buffer (PH = 7,4). PE-labeled antibodies in the amount of 20 mcl were added in the resuspended sediment to the intracellular marker *Foxp3*. This solution has been incubated for 30 min in a dark place at room temperature. Then the cells were twice washed with 2 mL of phosphate saline buffer (PH = 7,4).

Measurements were carried out on a cytofluorimeter FACSCalibur (Becton Dickinson, USA), which has a laser with 488 nm wavelength as well as standard filters. The analysis of the obtained data was performed by means of a software application BD Cell CellQuest for Mac OS X.

### 2.4. Determination of the Amount of CD3^+^CD4^+^CD25^−^ T Helpers in Peripheral Blood

To determine the level of expression of superficial receptors CD3, CD4, and CD25 in peripheral blood lymphocytes, we used the method of laser three-color flow cytometry with fluorescently labeled MCAB.

Staining of the markers CD3, CD4, and CD25 was conducted according to the protocol of the manufacturing company (“Becton Dickinson (BD),” USA).

**The Course of Work**

After isolation, mononuclear leucocytes were twice washed with phosphate saline buffer (pH = 7,4), every time having been resuspended and centrifuged for 10 min at 1500 rev/min. Then the supernatant was drained off, the remaining sediment was resuspended in phosphate-saline buffer, and the amount of cells was standardized in the suspension to 10 × 10^6^ cells/mL. To stain lymphocytes, we added 50 mcl of the mononuclear leucocyte suspension and 20 mcl of conjugated MCAB-CD3 (PerCP-Cy5,5)/CD4 (FITC)/CD25 (PE) (“Becton Dickinson (BD)”, USA, cat. No. 333170) to every cytometric test tube, mixed on vortex and incubated for 15 min in a dark place at room temperature.

Measurements were carried out on a cytometer FACSCalibur (Becton Dickinson, USA), which has an argon laser with 488 nm wavelength as well as standard filters. The analysis of the obtained data was conducted by means of a software application BD CellQuest for Mac OS X.

### 2.5. Statistical Processing of the Research Results

Analysis of the primary data was conducted using the methods of statistical description and verification of statistical hypotheses. All quantitative indicators were tested for normal distribution using Shapiro-Wilks test. For normally distributed samples, we calculated average sample characteristics: arithmetic average (*X*), mean square deviation (*σ*), and error of mean (*m*). For the samples which distribution differed from normal one we calculated median (*M*) as well as the first and the third quartiles (*Q*_1_, *Q*_3_).

When the feature in the researched samples corresponded with the normal law of distribution, verification of hypotheses about equality of average sample values was conducted using unifactor variance analysis. To evaluate certainty of differences of the sample numerical characteristics which are not subject to normal distribution, we used the Kruskal-Wallis test. For pairwise comparison of indicators in the researched groups, we used the Mann-Whitney test for independent groups. The difference in indicators in the compared groups was considered statistically significant at the significant level *P* ≤ 0,05. Calculations were performed using the program Statistica 6.0.

## 3. Results and Discussion

115 patients with advanced destructive forms of newly detected pulmonary tuberculosis (85 men and 30 women at the age of 18–55, average age 44 ± 12  years) have been examined. All patients were divided into 3 groups according to the clinical form of the disease: a group with infiltrative pulmonary tuberculosis (ITB) contained 65 people, with disseminated pulmonary tuberculosis (DTB) 31 people, with fibrous-cavernous pulmonary tuberculosis (FCTB) 19 people. When dividing the patients into groups, we took into account drug sensitivity of the causative agent to the basic ATD: the group of patients having MBT sensitive to basic ATD contained 68 people and the second group included 47 patients with MBT resistant to the first-line ATD (such as isoniazid, rifampicin, streptomycin, and ethambutol).

The control group included 26 healthy donors with similar sex and age characteristics (16 men and 10 women at the age of 18–55, average age 44 ± 12  years).

The carried-out research allowed us to conclude that the amount of T helpers in blood of TB patients is changing multidirectionally depending on the clinical form of the disease and sensitivity of *M. tuberculosis *to ATD. It has been revealed that the increase in the number of lymphocytes with immunophenotype CD3^+^CD4^+^CD25^−^ (T-helpers) is registered in the group of ITB patients (in comparison with the group of healthy donors), whereas in the groups of disseminated and fibrous-cavernous TB patients the number of CD3^+^CD4^+^CD25^−^ cells is decreasing (Table 1). Under TB with MDR-TB, the amount of T helpers is bigger in the group of ITB and DTB than in that of DS TB patients, whereas under FCTB the presence or absence of drug resistance of *M. tuberculosis* does not affect the amount of T helpers (Table 1).

It should be noticed that the above-stated sub-population of T lymphocytes is heterogeneous and forms the general idea of the amount of T helpers in blood of TB patients. Initially it was thought that T helpers are differentiated into two stable subpopulations: T helpers (Th) Th1 and Th2, the characteristic feature of which is production of immunoregulatory cytokines, multidirectional in their spectrum of action [16]. However, the research carried out in recent years allowed us to determine that among CD-4-positive T lymphocytes there are other cells which have “helper” functions. In particular, it was demonstrated that among CD4^+^ T lymphocytes there are Th17, Treg, and adaptive regulatory T cells: Th3, Tr1, and Foxp3^+^ Treg, which are formed in the course of immune response, as well as Th-activated cells and recently identified follicular T-helpers (T_FH_), whose function presumably lies in protection from extracellular pathogens [13, 17]. A peculiar feature of specific immune response under ITB is relatively adequate realization of Th1-dependent inflammatory response, the effector cells of which are CD4^+^ T lymphocytes. In connection with this, the increase in the number of CD4^+^ T-cells under ITB is viewed as a logical fact. At the same time the above-shown decrease in the total amount of T-helpers in blood of DTB and FCTB patients is the evidence of inhibition of clonal proliferation of CD4^+^ T cells (Table 1).

As for the patients with infiltrative and disseminated MDR TB, where the biggest amount of T helpers was detected in comparison with DS TB and healthy donors, is it likely that drug-resistant strains of MBT do not have a pathological effect on the process of T lymphocyte differentiation and proliferation under acute and subacute MDR TB, which include ITB and DTB? Nevertheless, to answer this question, further more profound studying of the subpopulation composition of T-helpers in TB patients is necessary.

**Figure 1:**
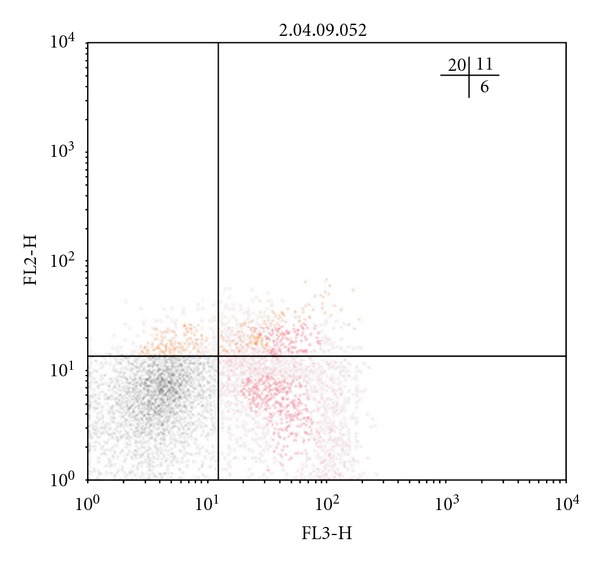
Individual bar chart of distribution of regulatory T cells in a population of CD4-positive blood lymphocytes, expressing CD25 and Foxp3, in a healthy person. Relative content of CD4^+^CD25^+^Foxp3^+^ cells (upper-right quadrant) 3,02%, CD4^+^CD25^−^Foxp3^+^ cells (upper-left quadrant)—6%, CD4^+^CD25^+^Foxp3^−^ cells (lower-right quadrant)—26%.

**Figure 2:**
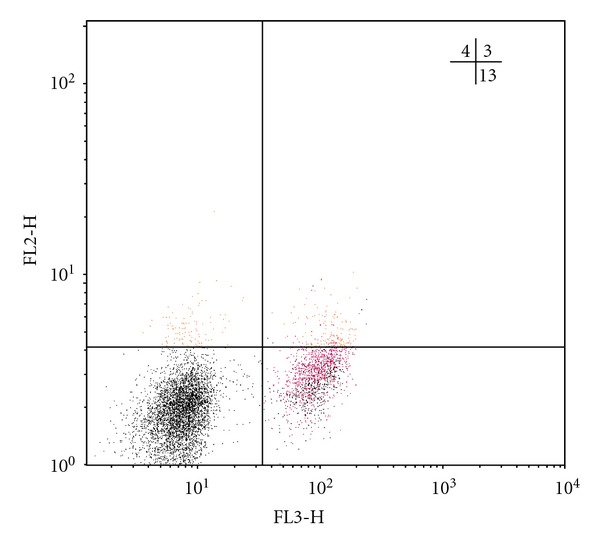
Individual bar chart of distribution of regulatory T cells in a population of CD4-positive lymphocytes of blood, expressing CD25 and Foxp3, in a patient with infiltrative pulmonary tuberculosis (patient N., 39 years old, diagnosed with infiltrative tuberculosis S_1-2_ of the right lung in a phase of decay and semination, MTB (+)). The relative abundance of CD4^+^CD25^+hi^Foxp3^+^ cells (upper-right quadrant) is 5%, CD4^+^CD25^−^Foxp3^+^ cells (upper-left quadrant) is 7%, CD4^+^CD25^+^Foxp3^−^ cells (lower-right quadrant) is 13.5%.

**Figure 3:**
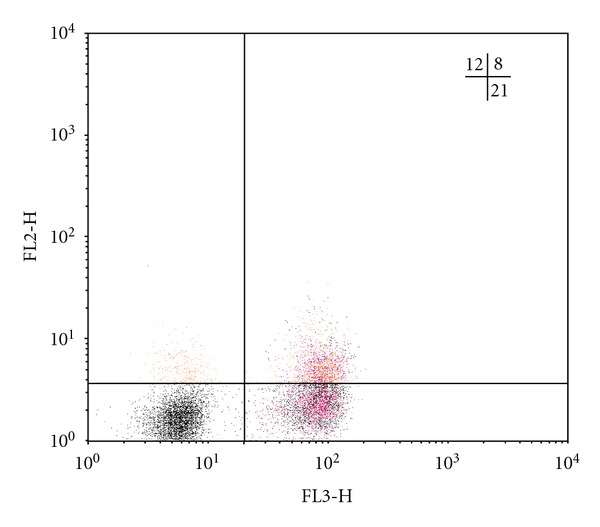
Individual bar chart of distribution of regulatory T cells in a population of CD4-positive lymphocytes of blood, expressing CD25 and Foxp3, in a patient with disseminated pulmonary tuberculosis (patient S., 28 years old, diagnosed with subacute disseminated pulmonary tuberculosis in the phase of infiltration and decay, MTB (+)). The relative content of CD4^+^CD25^+hi^Foxp3^+^ cells (upper-right quadrant) is 8%, CD4^+^CD25^−^Foxp3^+^ cells (upper-left quadrant) is 11,5%, CD4^+^CD25^+^Foxp3^−^ cells (lower-right quadrant) is 23.3%.

**Figure 4:**
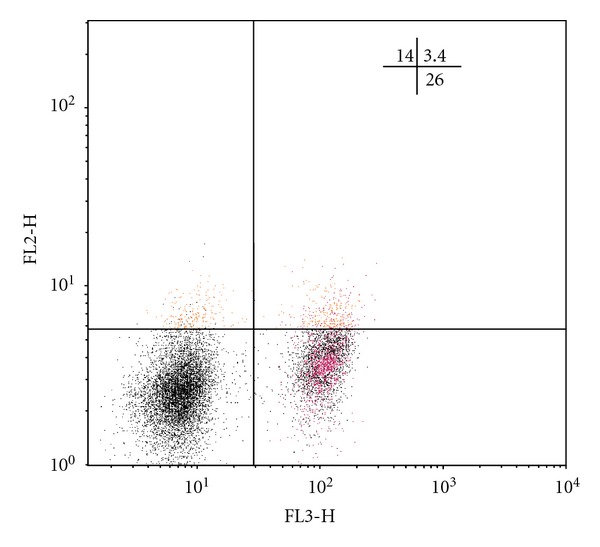
Individual bar chart of distribution of regulatory T cells in a population of CD4-positive lymphocytes of blood, expressing CD25 and Foxp3, in a patient with fibrous-cavernous pulmonary tuberculosis (patient S., 53 years old, diagnosed with fibrous-cavernous tuberculosis of the upper lobe of the left lung in a phase of infiltration and semination, MTB (+)). The relative content of CD4^+^CD25^+hi^Foxp3^+^ cells (upper-right quadrant) is 11%, CD4^+^CD25^−^Foxp3^+^ cells (upper-left quadrant) is 13,5%, CD4^+^CD25^+^ Foxp3^−^ cells (lower-right quadrant) is 25.3%.

As is known, natural Treg cells with the phenotype CD4^+^CD25^+^Foxp3^+^ and adaptive regulatory CD4^+^CD25^−^Foxp3^+^ T-lymphocytes are key suppressor cells of the immune response [13, 14]. The analysis of the amount of *Foxp3*-expressing regulatory T cells has shown that in all TB patients, irrespective of the clinical form of the disease, the increase in the number of CD4^+^CD25^+^Foxp3^+^ Treg is defined in comparison with the group of healthy donors, whereas the number of CD25-negative regulatory T cells, containing the *Foxp3 *molecule, is rising only in FCTB patients (Table 2). In Figures 1, 2, 3, and 4 Treg cells with the phenotype CD4^+^CD25^+^Foxp3^+^ are localized in the right upper quadrants and CD4^+^CD25^−^Foxp3^+^ cells in the left-upper quadrants.

 This fact allows us to suggest that in the case of chronic destructive process in Treg-mediated specific immunosuppression, regulatory T cells, both natural as well as formed, are involved in the course of the adaptive immune response.

 Tuberculosis, with the exception of acutely progressive forms, is considered to be “slow” infection, whose feature is long-flowing, but unfortunately, poor immune response against the background of abundance of a constantly persistent antigen. The previous studies we conducted have shown that dysregulation of cytokine production is met in patients with TB, and, in particular, the hypersecretion of immunoregulatory cytokines with suppressor activity (IFN*γ*, IL-4, IL-10, TGF-*β*) against the background of reduction of the secretion of IL-2 has been found out [18]. In this regard, as demonstrated in this study, the reduction in the number of T lymphocytes with the phenotype CD4^+^CD25^+^Foxp3^−^ in all clinical forms of TB (Table 2) is more likely connected with imbalance of cytokine production with a predominance of secretion of immunosuppressive cytokines and, consequently, the inhibition of not only proliferation, but also differentiation of Th0 into activated antigenspecific Th1-lymphocytes. In Figures 1, 2, 3, and 4 the CD4^+^CD25^+^Foxp3^−^ cells are localized in the right-lower quadrants. Interestingly, the fate of selection occurring in the thymus of *Foxr3*-negative CD4^+^CD25^+^ regulatory T cells by activation of endogenous (mitochondrial) apoptosis after their migration into the circulating blood and secondary lymphoid organs is further determined by different mechanisms of immune regulation, many of which are still not established [19, 20].

As our studies have shown, the number of CD4^+^CD25^+^Foxp3^+^ Treg lymphocytes in the blood of patients with ITB and DTB, regardless of the drug sensitivity of the causative agent to anti-tuberculosis drugs before specific therapy, that is, in the acute phase of tuberculous infection, has been rising with respect to the number of CD4^+^CD25^+^Foxp3^+^ Treg lymphocytes in healthy donors, as well as in general in the groups (Tables 2 and 3). A similar pattern was also observed in FCTB with MDR of the causative agent (Table 3).

 In our opinion, such changes primarily contribute to the formation of suppression of Th1-response to prevent the development of hyperergic immune responses and damage of lung tissue. For example, in the studies of Raghvan and Holgren [21] and Lee et al., [10] the deterrent role of Treg in the development of intensive immune inflammation and immune pathology accompanying various infectious processes has been shown [21, 22].

However, in the long run, this, to some extent, compensatory reaction associated with increased proliferation and differentiation of Treg, leads to negative consequences in the form of weakening of the effectiveness of protective immunity, promoting generalization and chronicity of infection [23].

 It is clear that increasing numbers of CD4^+^CD25^+^Foxp3^+^ Treg in blood of patients with TB are an unfavorable factor. The main way of realization of the effect of Treg is through the implementation of triple-contact interaction, in which, together with Treg and target cells, the tolerogenic dendritic cells (TDCs) are involved [13, 24]. TDC and Treg have mutually activating properties, presumably by contacting “cell-to-cell.” Perhaps, Treg prevents the formation of the immune synapse between TDC and effector T cell. It is assumed that the basis of this mechanism is competitive interaction of the negative activator CTLA-4 (*cytotoxic T-lymphocyte antigen-1*) with costimulatory molecules B7 (CD80/86) on the surface of target cells, promoting the formation of T-cell anergy [13]. Thus, an increasing number of Treg in almost all clinical forms and variants of the TB course lets us suggest that they carry out major immunosuppressive function in tuberculosis infection.

It is known that Treg cells with the phenotype CD4^+^CD25^+^Foxp3^+^ “are trained” in the thymus at the stage of negative selection and leave the thymus as the population of natural Treg, which has maximum suppressor activity. At the same time it is shown that a subpopulation CD4^+^CD25^+^Foxp3^+^ Treg is replenished in the peripheral section of immune system at the expense of their development from CD4^+^CD25^−^ T cells (the conversion of T-helpers into the regulatory T-cells) as a result of intercellular interactions with participation of costimulatory molecules with action of TGF-*β* and in the presence of TDC [13]. Conversion is a manifestation of the expression of the *Foxp3* molecule within a cell, as well as molecules CD25 and CTLA-4 on its surface [19]. In connection with the above-mentioned information, it is logical to assume that rather high number of adaptive regulatory T cells in the blood of patients with TB is caused not only by activation of intercellular interactions, but also by the enhanced production of cytokine-inductor Treg-TGF-*β* in pulmonary tuberculosis [18]. In addition, the source of CD4^+^CD25^+^Foxp3^+^ T cells could also be CD4^+^CD25^−^Foxp3^−^ T-helpers, which are differentiated in the thymus and which form a reserve pool for the formation of CD25^+^ Treg lymphocytes [11, 24].

Regulatory T cells with the phenotype CD4^+^CD25^−^Foxp3^+^ in the absence of expression on the surface of CD25 marker are considered to be induced, that is, generated on the periphery under the influence of TGF-*β*, as the expressions of the *Foxr3* marker largely contribute to TGF-*β* among all the cytokines suppressors [9, 25]. We found the increase in the number of CD4^+^CD25^−^Foxp3^+^ regulatory T cells in patients with MDR TB compared to drug-sensitive variant of the disease in ITB and FCTB patients (Table 3).

Perhaps this could be induced by the mycobacteria themselves. Thus, Sahno et al. [26] suggested that the strains of mycobacteria, resistant to standard chemotherapy, have special properties with respect to induction of CD4^+^CD25^+hi^ regulatory T cells with suppressor activity [26]. Thus, we cannot exclude the fact that in MDR TB, on the one hand, the activation of mechanisms of immunosuppression takes place due to both natural and adaptive regulatory T cells, and on the other hand, the drug-resistant strains of the MBT may contribute to suppression of immune response by means of induction of Treg.

As for the role of Treg in immunopathogenesis of drug-resistant TB, in this case it is difficult to underestimate it from the point of formation of the suppressor regime of immunoregulation in pulmonary TB in general. From this point of view the increase in the number of Treg at the periphery among patients with drug-resistant TB is also considered as a prognostically unfavourable factor leading to prolonged course of the disease as well as immunological discredit of the patient.

It is known that the main target cells of the influence of Treg are the activated CD4^+^ and CD8^+^ T cells, the key effector cells of antituberculosis immunity. Naive T cells are more sensitive to Treg than Th1 and Th2 lymphocytes [27, 28]. The identified reduced content of T lymphocytes with immunophenotype CD4^+^CD25^+^, which do not express the transcription factor *Foxr3*, in patients with TB, regardless of clinical forms of the disease and with no association with drug sensitivity of the MBT to anti-tuberculosis drugs, in comparison with the group of healthy donors (Tables 2 and 3), can probably be determined by the inhibition of proliferation and differentiation of T helpers, as well as by the influence of regulatory Foxp3^+^ T-cells with suppressor activity and which could affect the CD4^+^ and CD8^+^ T-lymphocytes by direct contact “cell-to-cell.”

At the same time, the reduction of CD4^+^CD25^+^Foxp3^−^ T lymphocytes in peripheral blood under TB can be considered as manifestation of T cell immunodeficiency associated with either the initial disintegration of the immune system in patients with TB, or with the formation of immune deficiency against the background of the spreading TB infection.

As mentioned above, *Foxp3*-negative T cells may be of thymic origin, as well as generated at the periphery. In addition, this subpopulation of lymphocytes is heterogeneous and, along with regulatory T cells, involves activated T helpers. Thus, we can assume that the decrease in the number of T lymphocytes with the phenotype CD4^+^CD25^+^Foxp3^−^ in blood in FCTB is determined by the deficiency of activated T helpers.

It is known, that in case Treg come into action by contact-dependent way towards the CD4^+^-cells, the target cells themselves gain suppressor functions: they gain the ability to secrete cytokines inhibitors, thus, inhibiting the proliferative and secretory activity of the secondary targets [29, 30]. In light of the shown changes and given the data from literature, it is obvious that the maintenance of the effective immune response in such a situation becomes impossible, which will inevitably lead to an unfavorable clinical course of TB.

## 4. Summary

1. The mechanism of immunologic deficiency, accompanying the course of pulmonary tuberculosis, is associated with the increase of *Foxp3*-expressing regulatory T cells with suppressor activity in blood, and is associated with the reduction in the number of CD4^+^CD25^+^Foxp3^−^ T lymphocytes (*Foxp3*-negative regulatory T cells and activated T helpers).
2. Imbalance of subpopulation composition of *Foxp3*-expressing regulatory T cells in patients with different clinical forms of multiple drug-resistant pulmonary tuberculosis is determined by higher content of CD4^+^CD25^+^Foxp3^+^ Treg in blood in the case of DTB and is combined with the increase in the number of CD4^+^CD25^−^Foxp3^+^ adaptive regulatory T cells in the case of ITB and FCTB.
3. In patients with ITB, including multidrug-resistant *M. tuberculosis*, an increased number of CD3^+^CD4^+^CD25^−^ T helpers is determined by the pathogenic features of the development of the tuberculosis infection and is connected with the activation of Th1-dependent immune response. Reduction in the number of T helpers in the blood of patients with DTB and FCTB mediates inefficient implementation of cell-mediated protective immunity.

## Figures and Tables

**Table 1:** Relative amount of T helpers (CD3^+^CD4^+^CD25^−^) in peripheral blood of patients suffering from pulmonary tuberculosis, depending on the form of the disease and sensitivity of the causative agent to antitubercular drugs (%), Me (*Q *_1_–*Q *_3_).

Type of a researched patients	Relative amount of T helpers in the group	Relative amount of T helpers in the group	
		depending on sensitivity of the causative	
		agent to antitubercular drugs	
		DS TB	MDR TB
Healthy donors	35,38 (32,74–38,74)		

Patients with ITB	45,47	38,20	54,41
	(43,07–47,82)	(33,04–47,88)	(54,38–54,89)
	*p*_1_ = 0,038		*p*_1_ = 0,042
			*p*_4_ = 0,036

Patients with DTB	31,61	23,90	39,38
	(29,33–34,41)	(16,84–26,66)	(24,57–48,75)
	*p*_1_ = 0,042	*p*_1_ = 0,043	*p*_1_ = 0,049
	*p*_2_ = 0,042	*p*_2_ = 0,041	*p*_2_ = 0,041
			*p*_4_ = 0,048

Patients with FCTB	25,46	24,4	26,0
	(21,38–32,86)	(22,38–25,35)	(21,20–28,70)
	*p*_1_ = 0,029	*p*_1_ = 0,037	*p*_1_ = 0,041
	*p*_2_ = 0,048	*p*_2_ = 0,045	*p*_2_ = 0,028
	*p*_3_ = 0,035		*p*_3_ = 0,039

*Note: p*_1_: the level of statistical significance of differences in comparison with the group of healthy donors; *p*_2_: in ITB patients; *p*_3_: in DTB patients; *p*_4_: in DS TB patients.

**Table 2:** Subpopulation composition of regulatory T cells of peripheral blood in patients suffering from pulmonary tuberculosis, depending on a clinical form of the disease (%), Me (*Q *_1_–*Q *_3_).

Groups of the researched patients	Relative content of regulatory T-cell subpopulations		
	CD4^+^CD25^+^Foxp3^+^	CD4^+^CD25^−^Foxp3^+^	CD4^+^CD25^+^Foxp3^−^
Healthy donors	2,63	5,12	25,45
	(2,00–3,29)	(4,76–9,75)	(22,30–27,60)

ITB patients	4,48	6,95	17,52
	(3,10–6,00)	(5,50–11,20)	(9,400–22,60)
	*p*_1_ = 0,047		*p*_1_ = 0,036

DTB patients	5,35	6,30	13,50
	(3,75–7,14)	(5,50–8,00)	(8,400–17,20)
	*p*_1_ = 0,014		*p*_1_ = 0,005
			*p*_2_ = 0,026

FCTB patients	4,80	8,50	19,50
	(3,20–6,00)	(4,20–11,50)	(13,400–24,30)
	*p*_1_ = 0,045	*p*_1_ = 0,049	*p*_1_ = 0,038
			*p*_2_ = 0,047
			*p*_3_ = 0,049

Note: *p*_1_: the level of statistical significance of differences in comparison with the group of healthy donors; *p*_2_: in ITB patients; *p*_3_: in DTB patients.

**Table 3:** Subpopulation composition of regulatory T cells of peripheral blood in patients suffering from pulmonary tuberculosis, depending on a clinical form of the disease and sensitivity of the causative to antituberculosis drugs (%), Me (*Q *_1_–*Q *_3_).

	Groups of patients	Relative content of regulatory T-cell subpopulations		
		CD4^+^CD25^+^Foxp3^+^	CD4^+^CD25^−^Foxp3^+^	CD4^+^CD25^+^Foxp3^−^
	Healthy donors	2,63	5,12	25,45
		(2,00–3,29)	(4,76–9,75)	(22,30–27,60)

ITB patients	DS TB	4,32	5,81	9,72
		(4,12–8,25)	(4,12–9,63)	(7,14–21,69)
		*p*_1_ = 0,007		*p*_1_ = 0,003
	MDR TB	5,08	8,41	16,27
		(3,12–7,42)	(5,62–11,58)	(9,13–25,92)
		*p*_1_ = 0,039	*p*_1_ = 0,005	*p*_1_ = 0,048
			*p*_4_ = 0,037	*p*_4_ = 0,017

DTB patients	DS TB	5,31	5,63	13,71
		(2,84–9,48)	(3,79–8,47)	(9,64–17,08)
		*p*_1_ = 0,015		*p*_1_ = 0,006
	MDR TB	4,42	6,75	13,98
		(3,17–7,53)	(4,82–8,16)	(7,52–17,80)
		*p*_1_ = 0,038		*p*_1_ = 0,006

FCTB patients	DS TB	2,72	3,25	19,62
		(2,09–3,66)	(2,17–5,82)	(11,31–31,75)
		*p*_3_ = 0,013	*p*_2_ = 0,014	*p*_3_ = 0,018
	MDR TB	6,82	12,27	17,68
		(3,17–10,42)	(8,45–13,09)	(12,42–23,19)
		*p*_1_ = 0,003	*p*_1_ = 0,017	
		*p*_4_ = 0,039	*p*_4_ = 0,015	

Note: *p*_1_: the level of statistical significance of differences in comparison with features of healthy donors; *p*_2_: in ITB patients; *p*_3_: in DTB patients; *p*_4_: in DS TB patients.
